# Snorkelling and Breath-Hold Diving Fatalities in Australia—A Review of 317 Deaths

**DOI:** 10.3390/ijerph22010119

**Published:** 2025-01-18

**Authors:** John M. Lippmann

**Affiliations:** 1 Australasian Diving Safety Foundation, Canterbury, VIC 3126, Australia; johnl@adsf.org.au; Tel.: +61-419-555326; 2 Department of Public Health and Preventive Medicine, Monash University, Clayton, VIC 3800, Australia; 3 The Royal Lifesaving Society—Australia, Sydney, NSW 2007, Australia

**Keywords:** breath-hold diving, cardiac arrhythmia, deaths, drowning, fatalities, freediving, snorkelling

## Abstract

As snorkelling and breath-hold diving are conducted in a potentially hostile environment by participants with varying skills and health, fatalities occur. In this study, snorkelling and breath-hold diving fatalities were investigated in Australia from 2000 to 2021 to identify causes and countermeasures. The Australasian Diving Safety Foundation database and the National Coronial Information System were searched to identify snorkelling/breath-hold diving deaths from 2000 to 2021. Relevant data were extracted, recorded, and analysed. The median age of the 317 victims was 48 years, two-thirds were overweight or obese, and almost half had health conditions, including ischaemic heart disease (IHD) and left ventricular hypertrophy (LVH), predisposing them to an arrhythmia-related snorkelling incident. One-third of victims were likely disabled by cardiac arrhythmias and at least 137 deaths were from primary drowning, with 34 following apnoeic hypoxia. Pre-existing health conditions, particularly IHD and LVH, predispose to many snorkelling deaths in older participants and may be somewhat mitigated by targeted health screening. Drownings from apnoeic hypoxia persist in younger breath-hold divers who should avoid pushing their limits without close monitoring. Skills practice in a controlled environment, increased focus on the importance of an effective buddy, and improved supervision are necessary to mitigate risk in the inexperienced.

## 1. Introduction

Snorkelling, including breath-hold diving, is a reasonably popular activity in Australia with an estimated 88,000 Australian residents aged 15 years or older participating annually [1]. In addition, the Great Barrier Reef (GBR) is one of the most popular international snorkelling destinations and has been so for several decades. A 2006 survey suggested that more than 500,000 international visitors participated in a total of more than 1.1 million snorkel dives in Queensland over a 12-month period, 94% of which were on the GBR [2].

Snorkelling participants include ‘surface snorkellers’ who are generally less experienced and are engaged in sightseeing, as well as ‘breath-hold divers’ (‘freedivers’), who are usually more experienced and hunting or harvesting seafood, playing competitive underwater sport, or practicing breath-hold in the ocean or pools. Surface snorkelling is particularly popular with the visitors to the GBR, and, more recently, to the Ningaloo region in Western Australia. Many of the participants are middle-aged or older overseas tourists for whom snorkelling on the GBR is an item to tick off on their “bucket list”.

Breath-hold diving, traditionally the domain of spear fishers, is becoming more popular as a competitive sport. The current world record for static apnoea is almost 12 min, and a freediver wearing fins and set weights has reached a depth of 131 metres unassisted [3].

**Figure 1:**
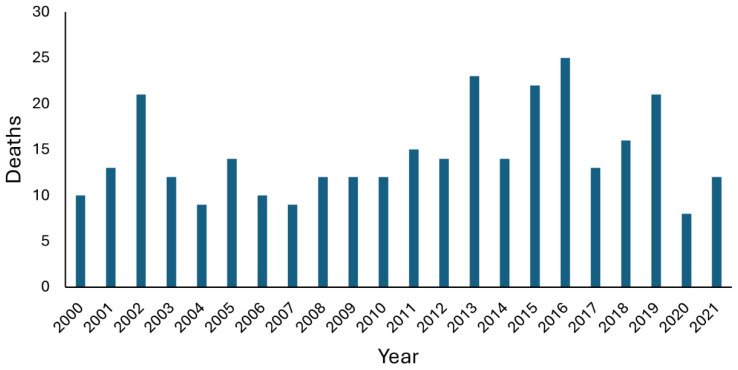
Annual snorkelling-related deaths in Australia, 2000–2021.

Given the nature of the environment and the variations in health, experience, and the activities of participants, some morbidity and mortality are inevitable, with annual fatalities shown in Figure 1.

Earlier reports from this on-going study reviewed data from 2001 to 2013 [4] and 2014 to 2018 [5] to determine the trends during those periods. In this report, additional earlier and subsequent data are utilised from the Australasian Diving Safety Foundation database [6] for which substantial coronial records were available to create a larger dataset, possibly the largest dataset of snorkelling-related deaths analysed to date. The aim of this study is to identify contributing factors over a 22-year period from 2000 to 2021 and assess existing and potential preventative strategies.

## 2. Materials and Methods

This study represents a retrospective case series of recorded snorkelling and breath-hold diving deaths in Australia from 1 January 2000 to 31 December 2021, inclusive. Ethics approval for the collection and reporting of these data was received from the Victorian Justice Human Research Ethics Committee to access the National Coronial Information System (NCIS) (approval number CF/21/18434). A search was made of the NCIS [7] for snorkelling and breath-hold-related deaths to 31 December 2021. Data obtained from the NCIS were matched with that listed on the Australasian Diving Safety Foundation fatality database (ADSF) [6] initially obtained via the media or word of mouth.

Relevant information on demographics, health, experience, dive circumstances, and forensic investigations were extracted and recorded. A chain of events analysis was performed for each case focusing on predisposing conditions, incident triggers, disabling agents, disabling conditions, and reported causes of death using existing templates with modifications to better tailor these to snorkelling incidents [8]. Most cases were reviewed by the ADSF Dive Fatality Research Group which included, among others, a forensic pathologist highly experienced in diving autopsies, a diving physician, a diving instructor, and a highly experienced dive fatality investigator.

Descriptive analyses based on means and standard deviations or medians and ranges, and t-tests, χ2 and Mann–Whitney tests for comparisons of age or BMI, as appropriate, were conducted using SPSS Version 29.0.0.0 (IBM Armonk, New York, NY, USA; 2022). The level of statistical significance assumed was *p* = 0.05 (two-sided).

## 3. Results

There were 317 recorded deaths with 198 (62%) victims likely to have been predominantly ‘surface snorkellers’ and 113 (36%) doing some ‘breath-hold diving’. Five individuals collapsed after exiting the water and there was insufficient information to determine the mode of diving in one.

### 3.1. Demographics and Characteristics

There were 278 (88%) males and 39 females. The median (IQR) age was 48 (33) years with a range of 15 to 85 years. Females were older than males (median ages 60 vs. 46 years) (*p* = 0.025). The breath-hold divers were younger than the surface snorkellers with median ages of 35 and 58 years, respectively (*p* < 0.001).

Autopsies were available in 293 (92%) cases with body mass index (BMI) recorded for 248 victims (220 males and 28 females). The mean (SD) BMI overall was 27.5 (5.9) kg·m^−2^ with a range of 16 to 66 kg·m^−2^. There was no significant difference in BMIs between the sexes (*p* = 0.742). One hundred and one (41%) of the victims were classified as overweight (BMI 25 to 29.9 kg·m^−2^) and 65 (26%) obese (BMI ≥ 30 kg·m^−2^).

The proportions of deaths between various states and territories were Queensland (56%), Western Australia (20%), New South Wales (14%), Victoria (7%), South Australia (2%), and the Northern Territory (1%). One hundred and forty (44%) victims were overseas tourists with a median (IQR) age of 60 (33) years. One hundred and sixteen (83%) of these overseas tourists were diving in Queensland and seventeen (12%) in Western Australia. Twenty-five (8%) victims were Australian residents from interstate and the remainder were Australian residents diving locally.

Forty-seven (15%) of the victims had never snorkelled before, seventy-six (24%) were inexperienced, and one hundred and four (33%) were reportedly experienced snorkellers. There was no indication of experience in 90 incidents. One hundred and eight (34%) were snorkelling with commercial operators (87% of these in Queensland with the vast majority being on the GBR) with the balance snorkelling privately. Two hundred and one (63%) victims were sightseeing, sixty-two (20%) were spearfishing, and thirty-four (11%) were harvesting seafood including abalone, scallops, and crayfish. Up to 34 (11%) drowned following apnoeic hypoxia from extended breath-holding, 8 of these while practicing breath-holding in pools, 6 with others nearby albeit not closely observing the victim. One hundred and two (32%) victims had set out snorkelling alone ‘solo’) and only seventy-nine (25%) were with someone at the time of their demise.

### 3.2. Predisposing Factors

Some factors can predispose to an adverse event while snorkelling. These include health-related factors such as poor fitness or a pre-existing medical condition, inexperience, adverse sea conditions, higher risk activities such as extended breath-holding without adequate supervision, seafood hunting, or harvesting in areas frequented by large sharks or crocodiles, among others. In many cases, there are multiple predisposing factors present which may have contributed to the incident. For example, an inexperienced snorkeller with a pre-existing cardiac condition who goes snorkelling in obviously unsuitable conditions has multiple vulnerabilities from the outset.

In this series, pre-existing health-related factors were identified as the most common predisposing factor and likely contributed to one hundred and fifty-one (48%) of the fatalities with many of the victims having multiple conditions. The most common were cardiac (112/151), particularly moderate to severe ischaemic heart disease (IHD, 95), left ventricular hypertrophy (LVH), and/or cardiomegaly (73) identified at autopsy. Fifty-six individuals had both substantial IHD and LVH/cardiomegaly. At least one hundred and one of these victims had received medical care for the identified or a related condition (e.g., hypertension), the remainder were undiagnosed. Thirty-three victims were being treated for hypertension (twenty-four of whom showed LVH and/or cardiomegaly at autopsy), eight for asthma, eight for diabetes, and six for seizures. Alcohol or drugs were contributing or causal factors in five fatalities.

Planning shortcomings, predominantly the decision to snorkel alone, distant from a buddy, or to set out in obviously adverse sea conditions likely contributed to one hundred and thirty-five (43%) incidents. Poor skills and inexperience were identified as likely contributors to at least eighty-three (26%) deaths. Higher risk activities predisposed to forty-seven (15%) of the deaths. These included extended apnoea (with or without hyperventilation) with inadequate supervision which led to apnoeic hypoxia and subsequent drowning in up to thirty-four incidents. Nine died while spearfishing in areas frequented by large marine predators, six were attacked by sharks, and three by crocodiles. One breath-hold diver suffered a cerebral arterial gas embolism after diving down and breathing from a friend’s scuba unit before ascending, obviously without adequate exhalation.

### 3.3. Disabling Conditions and Causes of Death

The *Cause of Death* is that reported by the forensic pathologist and subsequently by the coroner (Table 1). ‘Other’ includes two deaths attributed to stroke and one each to cerebral arterial gas embolism, fatty liver disease, and olanzapine toxicity. Trauma deaths resulted from shark attacks (9), boat impacts (5), crocodile attacks (3), stingray injury (1), and blunt force trauma after impacts with rocks in rough seas (1). ‘Unknown’ refers to deaths where no body was found and ‘Unascertained’ is where a cause was unclear as reported by the forensic pathologist/medical examiner.

The *Disabling Condition* is defined as an injury or condition directly responsible for death, or for incapacitation followed by death from drowning [8]. For example, if a snorkeller suffers a cardiac arrhythmia, becomes unconscious and subsequently drowns, the disabling condition is identified as cardiac-related, but the cause of death would generally be reported as drowning (which was in fact secondary). Close examination of the likely chain of events in cases in this series and the associated autopsies suggests that the likely disabling condition for one hundred and thirty-seven (43%) of the victims was primary aspiration of water (“asphyxia” or “primary drowning”), and a cardiac event in one hundred and eleven (35%). In another eighteen cases, it is unclear whether the disabling condition was asphyxia or cardiac as there was equivocal evidence for both. Immersion pulmonary oedema (IPO) was identified as the likely disabling condition in four incidents, although there may have been more as it can be very difficult to distinguish from drowning unless the incident was clearly witnessed and/or there is a previous history of dyspnoea with immersion.

Surface snorkellers were more likely to be disabled by cardiac event than were breath-hold divers (50% vs. 15%), and less likely to be disabled by primary drowning (41% vs. 61%). There was a substantially higher association of cardiac disabling conditions in snorkellers with identified predisposing health conditions than in those without (OR = 15.68; 95% CI = 8.83 to 28.48, *p* < 0.001).

## 4. Discussion

Victims were predominantly males from one of two demographic cohorts. The first were relatively young, healthy, and experienced breath-hold divers who often succumbed to primary drowning, in many cases subsequent to apnoeic hypoxia. The second were aged over 50 years and were frequently overweight and inexperienced snorkellers with pre-existing medical conditions, diagnosed or occult. These conditions predisposed them to a snorkelling-related mishap, often associated with a cardiac arrhythmia.

Pre-existing medical conditions, cardiac and otherwise, have been linked with drownings in a variety of aquatic activities [9,10]. Cardiac arrhythmias can be precipitated by immersion or submersion per se in which buoyancy counters the effect of gravity, encourages redistribution of venous blood from the limbs to the thorax and results in a substantial increase in the cardiac workload [11,12,13]. The workload is further increased during snorkelling from exercise, anxiety, cold-induced vasoconstriction, respiratory resistance, and elevated heartrate, increasing the likelihood of a cardiac event in a predisposed individual. In addition, apnoea, coupled with the mammalian diving reflex, can precipitate cardiac arrhythmias, especially in cooler waters and even in young, healthy individuals [14,15].

Given the lack of a definitive postmortem test to determine whether an arrhythmia has occurred, medical history, witness reports, and autopsy evidence of significant cardiac disease or abnormality are used to assess the likelihood of its occurrence. Arrhythmias are commonly associated with coronary atherosclerosis with a stenosis likely to result in ischaemia generally regarded as being greater than a 75% narrowing of the lumen [16]. However, a substantially smaller stenosis may be significant when associated with other potentially mitigating factors such as left ventricular hypertrophy, which is a known risk factor for sudden cardiac death and an increased incidence of serious arrhythmias [17,18,19].

The high prevalence of moderate to severe ischaemic heart disease, often in combination with left ventricular hypertrophy and/or cardiomegaly, together with the multiple cardiac stresses associated with snorkelling, suggests that cardiac arrhythmias were the likely precipitant of many of these, often silent, deaths.

**Figure 2:**
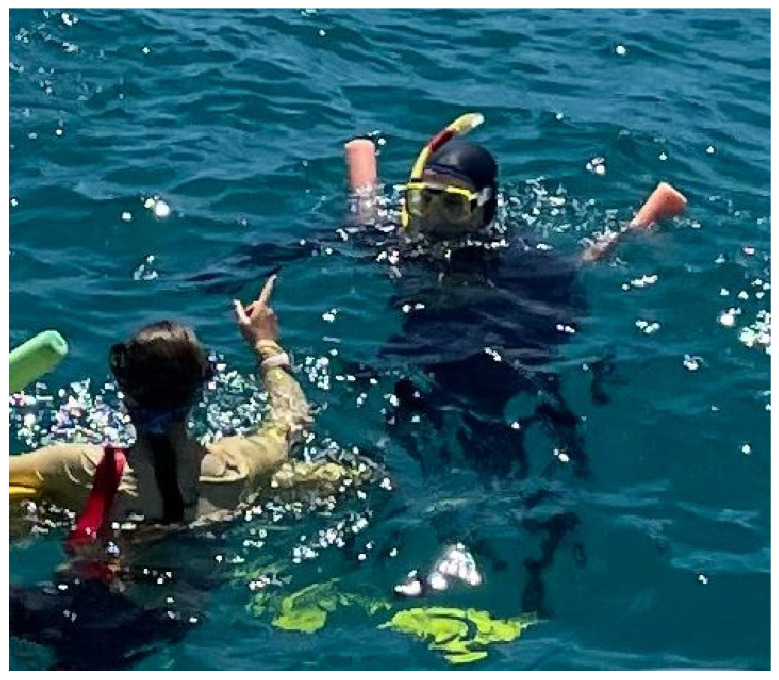
An “at risk” snorkeller with a marked snorkel, buoyancy aid, and close supervision.

Because of the potential for a cardiac event while diving, scuba divers aged 45 years or over are advised to undergo a diving medical examination with a focus on cardiovascular assessment [20]. Since many of the potential cardiac triggers described above are common to scuba divers and snorkellers, it seems prudent that older active or potential snorkellers should also discuss their cardiac health with their doctors, who, ideally, would be cognisant of the associated risk and consider what investigations, if any, might be appropriate. This can be an important part of pre-travel preparations if snorkelling is planned or a possibility. Those with a known pre-existing medical condition who are on a snorkel excursion with a commercial operator should ensure they declare this to the operator so that risk mitigation procedures can be implemented by a conscientious operator. These may include an identification marker such as a coloured tag on the snorkel, closer supervision, and possibly a floatation aid (Figure 2).

Drowning has traditionally been recorded as the default cause of death of a person recovered from water with some, often non-specific, pulmonary changes and no other obvious cause of death. The differences between the 61% with drowning as the cause of death and the 43% with asphyxia as the disabling condition in this series, and, similarly, between the relative cardiac proportions, may largely reflect the cases where drowning was secondary to a cardiac arrhythmia. When analysing snorkelling, scuba diving, and swimming fatalities, it is often very informative to identify the likely disabling condition as it can provide clearer insights into the chain of events leading to death and so help to identify appropriate mitigation strategies.

Another increasingly discussed cause of morbidity and mortality is immersion pulmonary oedema (IPO) which has been reported in swimmers, surface snorkellers, breath-hold divers, and scuba divers [21,22,23,24]. It is often characterised by dyspnoea, cough, frothy and often blood-stained expectoration, fatigue, and hypoxia. At autopsy it is very difficult, usually impossible, to distinguish IPO from drowning so witness reports and details of any previous episodes should be sought to increase confidence in the diagnosis. However, in the absence of these and with evidence of potentially arrhythmogenic cardiac disease at autopsy, cardiac factors are often more convincing, although left ventricular hypertrophy can also predispose to IPO [25].

The younger cohort of victims in this series was generally involved in breath-hold diving, involving spear fishing, and sometimes practicing extending their breath-hold limits. Drownings following apnoeic hypoxia persist despite the on-going efforts to raise awareness of the pitfalls of pushing limits, especially when alone, or effectively alone in the water, be it in the ocean or a pool without direct and close supervision. Although pre-dive hyperventilation is still practised by some, it is reported less frequently, having been largely replaced by various other apnoea-extending techniques such as specific training to better tolerate hypercapnia and hypoxia. However, rapid unconsciousness remains an ever-present threat, particularly during ascent but also in shallow swimming pools, and it is important to have a vigilant and capable rescuer immediately at hand who is ready and able to promptly rescue the diver and provide resuscitation and supplemental oxygen if indicated.

In this series, nine of the twelve snorkellers who were killed by sharks or crocodiles were spearfishing. More than 70% of all divers killed by sharks in Australia between 1960 and 2017 were hunting or harvesting seafood or near to where others were fishing [26]. This risk needs to be mitigated where possible by appropriate planning, including choice of dive site and keeping one’s catch distant. Boat impact injuries can be reduced using a ‘Diver Below’ flag, careful selection of dive sites, and the improved education and vigilance of boat operators.

**Figure 3:**
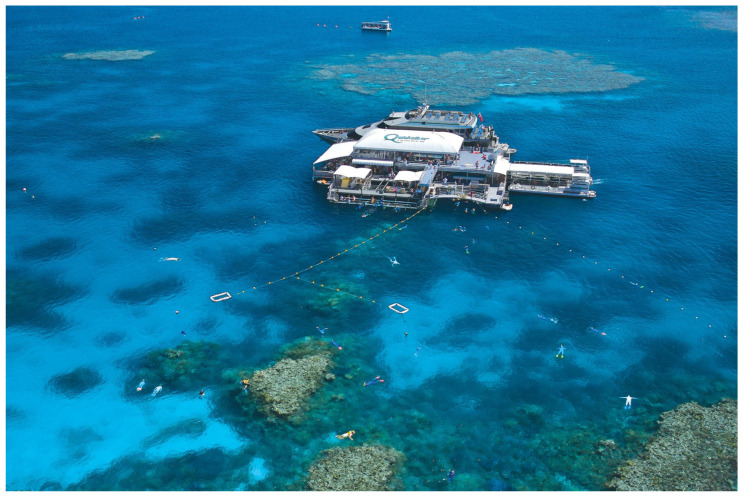
A large commercial snorkelling operation on the Great Barrier Reef. Of note, there is a marked snorkel area, rest stations, and several lookouts at multiple vantage points.

Inexperience is another major contributor to snorkelling-related deaths and is especially prevalent in tourists snorkelling on the northern reefs. Many victims are weak swimmers and novice snorkellers who are prone to aspirating water through their snorkels and/or masks, which can lead to laryngospasm, unconsciousness, and subsequent drowning. All snorkellers should adhere to the ‘buddy system’, but this is more important with the inexperienced who need to be monitored very closely. Buoyancy aids such as vests and noodles can provide support but do not always prevent drownings. Lookouts are common in commercial settings in Australia (Figure 3) but are sometimes required to oversee too many snorkellers, possibly in difficult conditions. This can lead to substantial delays before a distressed or unconscious snorkeller is noticed and rescued, with such delays greatly reducing the likelihood of survival.

Commercial snorkel operators should have appropriate first aid equipment, including oxygen equipment and automated external defibrillators, and staff trained in their use. In Queensland, where diving-related tourism is an important income source, there is a regulated Code of Practice that specifies requirements for screening, supervision, and the conduct of snorkel and scuba dives [27]. This has undoubtedly reduced the potential morbidity and mortality and can provide an example and valuable guidance for snorkel operators elsewhere, especially in some developing countries where snorkelling is becoming increasingly popular, accidents more frequent, and expert rescue and medical support less available.

### Limitations

As with any uncontrolled case series, the collection and analysis of the fatality data are subject to inevitable limitations and uncertainties associated with the investigations. Given that many incidents were unwitnessed, some of the assertions, such as the disabling conditions and, to some extent, the causes of death can be somewhat speculative.

## 5. Conclusions

Pre-existing health conditions, particularly ischaemic heart disease, often in combination with left ventricular hypertrophy, predispose to many snorkelling-related deaths in older participants and such deaths are usually cardiac-related. The focused health screening of older active or prospective snorkellers may reduce this mortality. Drownings from apnoeic hypoxia persist both in the ocean and in pools, often with others nearby. Breath-hold divers should avoid pushing their limits without being closely watched by a ready and capable rescuer. Inexperience and the lack of a buddy or an effective buddy system or supervision are strongly associated with fatalities. Skills practice in a controlled environment, redoubled education on the importance of an effective buddy, and improved supervision are necessary to mitigate risk.

## Figures and Tables

**Table 1:** Causes of death in 297 fatal snorkelling incidents.

Cause of Death	n (%)
Drowning	193 (61)
Cardiac	82 (26)
Trauma	19 (6)
Other	5 (1)
Unascertained	9 (3)
Unknown	9 (3)

## Data Availability

The data on which this study was based are only available from the National Coronial Information System with appropriate ethics approvals. The datasets presented in this article are not readily available because they are largely taken from restricted coronial documents which can only be accessed with particular ethics approvals. Requests to access such data should be directed to the Australian National Coronial Information System (ncis.org.au) (accessed on 10 January 2025).
